# Dynamic Clinical Algorithms: Digital Technology Can Transform Health Care Decision-Making

**DOI:** 10.4269/ajtmh.17-0477

**Published:** 2017-11-06

**Authors:** David Bell, Noni Gachuhi, Nassim Assefi

**Affiliations:** Intellectual Ventures Global Good Fund, Bellevue, Washington

## Abstract

Most health care in low-income countries is delivered at a primary care level by health workers who lack quality training and supervision, often distant from more experienced support. Lack of knowledge and poor communication result in a poor quality of care and inefficient delivery of health services. Although bringing great benefits in sectors such as finance and telecommunication in recent years, the Digital Revolution has lightly and inconsistently affected the health sector. These advances offer an opportunity to dramatically transform health care by increasing the availability and timeliness of information to augment clinical decision-making, based on improved access to patient histories, current information on disease epidemiology, and improved incorporation of data from point-of-care and centralized diagnostic testing. A comprehensive approach is needed to more effectively incorporate current digital technologies into health systems, bringing external and patient-derived data into the clinical decision-making process in real time, irrespective of health worker training or location. Such dynamic clinical algorithms could provide a more effective framework within which to design and integrate new digital health technologies and deliver improved patient care by primary care health workers.

## THE DIGITAL REVOLUTION AND PUBLIC HEALTH IN LOW-INCOME COUNTRIES

Two of the greatest obstacles to high-quality primary health care in low-income countries are a lack of skilled health workers and the limited access to reliable, actionable health information. Even where clinicians exist with sufficient skills, health information systems are rarely organized to collate and deliver feedback on health trends to the provider. Medical records have minimal content and may be difficult to access, and diagnostic results may arrive too late to influence the therapy.^1–3^ Communication gaps between health providers caring for the same patient result in fractured care. Barriers, such as limited capacity to collect and use data or accountability for performance, can severely limit the ability of health workers to integrate local epidemiology and real-time data on diseases.^4^ Although systems are moving toward digital transfer and collation of data centrally, processes that feed back to influence management are often cumbersome and restricted by rigid policies and guidelines. These barriers, which within the context of an already inefficient paper-based system, reduce the benefit patients might gain from data on current conditions (such as seasonal prevalence, disease outbreaks, and drifts in pathogen prevalence and drug susceptibility).

In contrast to the modest integration of digital technology into public health—especially the mobile phone and related applications—there has been a transformational improvement in digital technologies in other spheres such as mobile money and cash transfers in Kenya; the transportation sector through ride share programs such as Uber and Lyft in the United States and mobile commerce in retail. According to the International Telecommunications Union, more than five billion mobile phones have been sold in low- and middle-income countries, and mobile phones are available to more than 95% of the world’s population.^5^ Indeed, a significant proportion of the low-income populations targeted by United Nations Sustainable Development Goals will make use of mobile technology to access information through the internet and to tap into the financial services which previously had been out of bound to most people. Digital information systems are expanding in the health sector through localized electronic medical record (EMR) pilots and projects to improve patient recall for specific purposes such as HIV/TB service delivery and maternal and child health.^6,7^ At national levels, the District Health Information Software (DHIS2), which is now in use in more than 30 countries in Asia, Africa, and Latin America, is increasingly used to support data collation and reporting of trends and national health indices, and is beginning to be incorporated into EMR-level applications such as DHIS2 to more directly retrieve data.^8,9^ Gains from integrating data from EMR into DHIS2 for reporting purposes was demonstrated successfully in a study in Kenya, by reducing the amount of time that health workers spent manually transcribing the data as well as the completeness and accuracy of the data.^10^ Expanding on this capability to include real-time feedback could be immensely beneficial to patient care and outcomes. Data flow, however, tends to be one-way, and a comprehensive approach to use collated data couple with real-time data acquisition to support routine provider’s decision-making in a clinical context is lacking.

There are good empirical arguments for believing that advances in fields such as biometrics, connectivity, diagnostics, and artificial intelligence (AI) will translate into improvements in the quality of health care. Many studies in low-resource health systems have tested computerized clinical decision support systems (CDSSs), which link patient information—whether queried through electronic health records or manually entered—with evidence-based knowledge through rule- or algorithm-based computer software.^11–18^ A comprehensive review of early CDSS in 2005 assessed 100 controlled trials on patient outcomes—including diagnosis, preventive screening, clinical management of specific diseases, and drug prescribing and found improvement in most of the health providers (64%), whereas close agreement with trained nurses has been recently demonstrated using neural network-based learning algorithms.^17,19^ Some of the relevant factors in a CDSS that contribute to improved clinical outcomes are computer-generated decisions and a system that provides time and location recommendations to the provider about what to do.^20,21^

Innovative data systems that recognize and respond to epidemiological and management trends could address shortcomings of decision-making in primary health care—especially the use of quality data to inform clinical decisions. According to the World Health Organization, there was a shortage of 7.2 million health providers in 2013, projected to grow to a 12.9 million deficit by 2035 if current trends continue. This shortage is further aggravated by the quality of medical training received, insufficient human resources to train, poor facilities, and outdated and disease focused curricula.^22^ To help counteract the deficiency of physicians, particularly in rural and remote areas, community health workers (CHWs) and mid-level providers are often deployed as physician extenders. The great variability in the baseline educational levels and training of these CHWs from low literacy and 2 weeks of training in Botswana to high school diplomas and 2 years of training in Iran has an impact on the quality of the health care that they deliver.^23^

This article explores the future of computerized CDSSs for primary health care in low-resource settings. We suggest that advances of CDSSs in various settings, using the growing availability of big data in clinical decision-making, open up a radical technological approach that could enable countries with poor health infrastructure to capitalize on the information revolution transforming other sectors of society.^24^ By integrating clinical and local epidemiologic data with improved medical record systems, and improving digital linkages within and between clinics, a system that employs dynamic clinical algorithms (DCAs) could optimize the clinical care of patients, incorporating relevant data from all levels of the health system into decision-making in primary care consultation. Utilization of such data could improve the clinical decisions that health workers serving at the periphery of the health system could make. Although setting up such a process takes time, establishing a framework would provide a structure within which to cultivate technology and systems to realize this goal. Through task shifting and redistribution of service delivery, a CHW in a rural setting, equipped with a mobile phone or tablet, linked to consolidated data and DCAs, could provide a far higher standard of service.

## A CONCEPTUAL FRAMEWORK FOR DATA DRIVEN CASE MANAGEMENT

**Figure 1.**
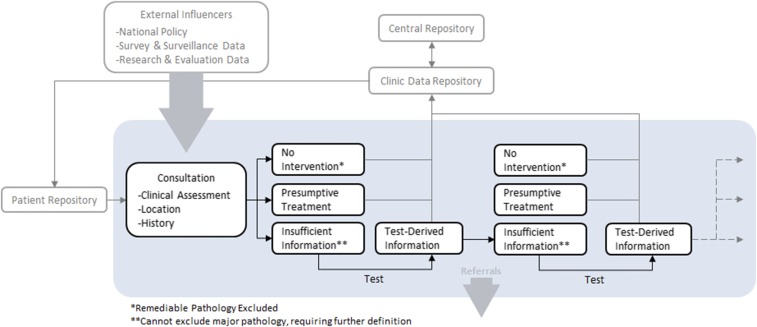
Basic components of a dynamic clinical algorithm. As with any clinician–patient consultation (shaded box), management is an iterative process with information gained at each step guiding subsequent decision-making. Each decision is potentially influenced by external factors as well as consultation-derived and patient-derived factors. Any highly trained and informed clinician will operate in this way to some extent. Introduction of improved communications, retrievable databases, and patient recognition, together with algorithms that operate with a degree of machine learning within certain set clinical bounds, could enable this level of decision-making to be available to communities where high-level clinician support is not available. This figure appears in color at www.ajtmh.org.

To transform medical care in low-income countries into a data-driven, logical, and optimized decision-making process, a DCA system will require, at minimum, an accurate means of patient identification, digitized health care information, and a connection with referral, specialized and supervisory levels of the health care system. A DCA system would link patient data to the decision-making process through three broad mechanisms (Figure 1). First, it requires a universal, de-identified clinical database that integrates local epidemiologic data (outbreaks, antimicrobial susceptibility, seasonality, and other historic and geographic trends), with health policy changes, using a search engine in near real time. A second level retains pertinent patient-specific background data (medical history, current medications, demographics, etc., known as EMR) linked to a biometric signature. Monitoring the data of patients who are mobile could be linked to such a system.

Algorithms, improved by prior cases of sufficient similarity that are instructive to the current case, would populate the patient’s clinical management path in real time with recommended next steps such as treatment, further diagnostics, or referral. Thirdly, every time a patient is treated, their anonymized data and medical course would get stored in the database to create a feedback loop that makes the system smarter (known as machine learning or deep learning) to guide the care of subsequent patients. DCAs could be developed to factor the weight of each feature being matched against an outcome of interest, as well as the relative value of (and permissible missing values for) the interacting data elements in the match process. Such algorithms could be modifiable to meet the changing terrain of infectious diseases and evidence-based medical practice.

This framework is broad and can address preventive care, diagnostics, therapeutics, and comprehensive disease management. Theoretically, DCAs can be geared toward different cadres of health workers and their settings and available infrastructure. For maximal efficacy, DCA systems should be developed on human-centered design principles (Table 1), which take into account the user of the system and involve them from the start in the development of the system. DCAs have the potential to affect the quality—the effectiveness, efficiency, economics, and safety—of health care.

**Table 1:** Principle components of a dynamic clinical algorithm system


1. Accurate patient recognition (biometrics).
2. Patient assessment & history; queried from other clinical information systems and transmitted from digital devices (medical record).
3. Patient diagnostics: system integration with digital medical diagnostic and monitoring devices (pathology).
4. Privacy and confidentiality.
5. Near or real-time localized infectious disease surveillance data.
6. Open-source software with multiplatform flexibility (to enable local adaption and integration to additional systems).
7. National government policies and guidelines.
8. Capacity of the system to work offline with easy return to connectivity once established.
9. Rugged hardware for rural and low-resource settings (portability, battery life etc.)
10. Minimal disruption of clinical workflow (limit the extra burden on health worker time).
11. User-friendly and culturally appropriate design interface.

## ADDING DYNAMIC ELEMENTS TO CLINICAL ALGORITHMS

**Figure 2.**
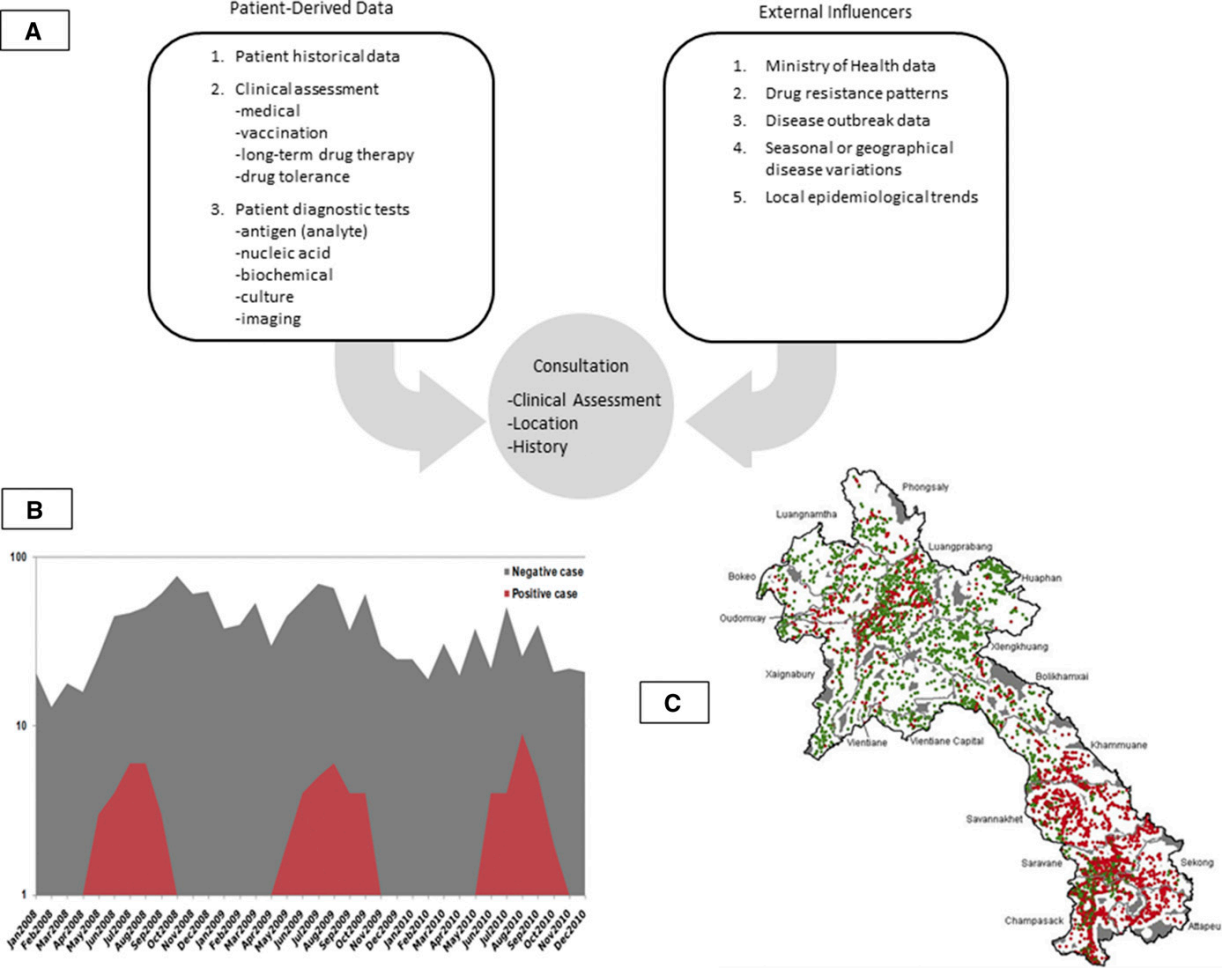
Examples of data that should influence the course of a clinical decision-making process. Through use of a dynamic clinical algorithm, these impact management without the clinician being aware of their existence, by increasing the weight of certain management options. (**A**) Patient-derived and external factors. (**B**) Example of seasonal disease prevalence. Clearly, the use of a dengue diagnostic test for investigation of fever should vary with the time of year (LOMWRU, MoH, Lao PDR, 2011). (**C**) Geographical variation of malaria incidence across relatively small distances in the Lao PDR should influence the choice of using a malaria diagnostic test on initial presentation for fever.^26^ This figure appears in color at www.ajtmh.org.

In low-income countries, infectious diseases, often in conjunction with underlying nutritional and physical vulnerabilities, are a major cause of morbidity and mortality. The risk of disease often depends on local prevalence, which is a factor of many elements including season, living conditions, environmental change, animal vectors, and population immunity. Diseases such as dengue or malaria, for instance, should only be considered a high probability during certain seasons, and health worker’s responses, including diagnostic testing, should be informed by epidemiology and logically reflect this (Figure 2). Current generations of real-time surveillance systems, which often use mobile health technology, offer the possibility of improving clinical decision-making by incorporating the most up-to-date information about local disease incidence into diagnostic algorithms.^25^ Although a deterministic human-driven approach could initially be necessary for the system to be acceptable, it is reasonable to assume that future improvements in data quality at input and acquired confidence in electronic systems could enable AI-driven algorithms to recognize patterns and automate changes that would appear as recommendations on a health workers’ screen without direct human intervention.

Incorporating real-time, epidemiologic data with patient history, diagnostic results and latest evidence-based clinical data, medication resistance, and supply chain issues into treatment plans for presenting patients makes these computerized models of clinical decision-making dynamic rather than the static, paper-based algorithms. DCAs take into account these ever-changing components when making decisions about intervention and treatment options rather than a reliance on nonchanging and static algorithms developed at a different time and place. The proliferation of mobile phones and increased access to the internet means that data can continuously flow back and forth between systems and users. This dynamic approach can be thought of as a kind of personalized, public health medicine, where the epidemiologic milieu and local treatment possibilities influence outcomes.

## TECHNOLOGY BUILDING BLOCKS REQUIRED

Responsive AI-based management algorithms will require the expertise of data inputters, data architects, data mining specialists, predictive modeling, and machine learning experts, and user interface designers to create engaging, intuitive software and robust novel algorithms that meet the needs of a changing health environment. Clinical, public health, and engineering teams will need to collaborate to generate algorithms that consider patient data matched against the outcome of interest, as well as the relative value of interacting data in the match process. These algorithms should be modifiable to reflect the changing nature of medicine and the dynamic nature of the data and use only the requisite patient data as to not overburden the health worker’s workflow. Of critical importance to the effectiveness of computerized algorithms is an automated function, which automatically prompts the user to use the system, and in addition, suggests the clinical action that the health worker should take. The algorithms and design would require localization—at a minimum to each region, language, facility and cadre of health worker. Finally, the system will need to work within certain parameters of variability set by the central health authority and be open to direction from an experienced on-site or remote clinician as health authorities, experienced clinicians, and patients will not readily accept handing over full decision-making power to a machine.

All the mechanisms guiding DCAs will need to be developed in close collaboration with clinicians and clinical policy makers. They require integration with technology improvements and adaptions in biometric recognition, connectivity, data retrieval, together with neural network or machine-learning algorithms that can adapt clinical decision-making within parameters considered clinically safe and appropriate to the clinical and cultural context.^17^ In particular, they will require a willingness to shift and accelerate decision-making processes within health systems—a process that will require political will and regulatory change.

## THE ROLE OF BIOMETRICS

**Figure 3.**
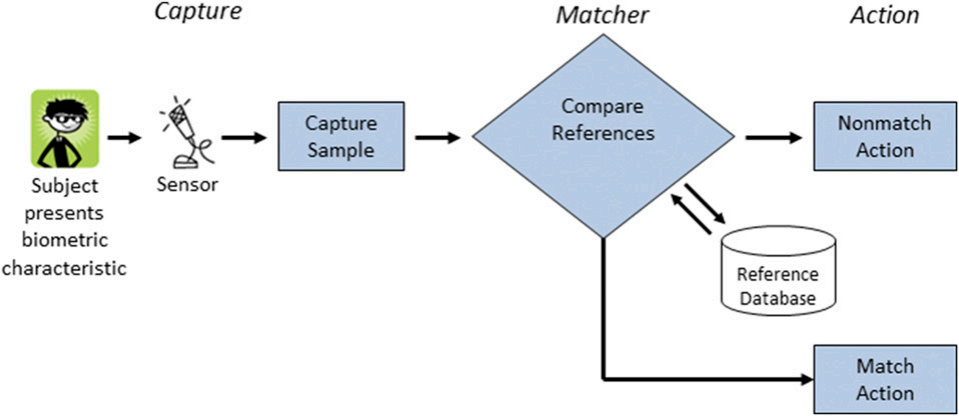
A general biometric recognition system. The biometric scan must first capture and store the reference biometric sample. On subsequent consultations, a new biometric sample (“matcher”) is compared with the reference sample database. An “action” step then follows: a decision based on matching or nonmatching. A plan must be in place to deal with failure to capture relevant biometric data.^27^ This figure appears in color at www.ajtmh.org.

Accurate patient identification and consistent linkage of patient data into the population health database are fundamental to the underlying operating assumptions of a successful DCA system. Biometric recognition technology is a promising way of identifying individuals in countries with inconsistent record keeping and patient tracking, including mobile populations of internally displaced people and migrants. The source of biometric identification is the patient’s unique anatomical, physiological, and/or behavioral features, such as fingerprint, face, iris, retina, palm, ear geometry or acoustics, vein pattern recognition, gait, odor, electrocardiogram, signature, and voice.^27^ Identification occurs by comparing a biometric sample obtained from the subject with a set of records stored in a database (Figure 3). Unlike usual identification methods based on patient documents (a passport, identification card, bank card, etc.) or memory (personal identification number, Social Security Number, password), biometrics allows for rapid identification of a person independent of patient or health worker recall and can be consistent across time.

Thus far, biometric technology is best known for assisting governments and private companies to verify identity at borders and police stations, provide better control of access to physical facilities and computer accounts, prevent fraud in insurance claims and social services, and manage security in institutions such as banks or on digital devices.^27^ The field is advancing rapidly, and there is great potential to use this technology in the health care context. India for example, has a large national biometric database to manage social security access. Known as the Aadhaar program, this Unique Identification Authority of India is using iris and fingerprint verification to identify all 1.2 billion citizens. By March 2017 (6 years into the project), over 1.1 billion people were scanned and assigned a unique identification number demonstrating the scalability of biometric technologies.^28^

As with any new technology, the effectiveness of biometric recognition depends as much on the social and public policy context as it does on the underlying technology, and to be fully accepted, the biometric system’s security, privacy, and legal goals need to be made explicit with minimal risks to the user and must not impede clinic workflows.^27^ However, biometric recognition and linking of personal data is a rapidly evolving field and opens a path for low-resource health systems to use digital technology to rapidly build the framework that is necessary to achieve more patient-centered care.

## CONCLUSION

The wave of rapidly evolving digital technology sweeping other industries presents an opportunity to create evidence-based, health care algorithms, where decisions are made with the input of solid data, predictive analytics, and documented outcomes, rather than on individual experience and inconsistent use of applicable informational resources and norms. DCAs could eventually empower the frontline health worker, providing an augmented ability to diagnose and treat diseases based on evidence and changing conditions and contribute to bridging the huge disparities in health care that currently exist. Although such a transformation will take time, the necessary components already exist and have already penetrated other sectors of society. The global health community has an opportunity to shape that transformation today and speed its delivery through a thoughtful and comprehensive approach to designing and delivering innovative health management systems.

## SUMMARY

1. DCAs based on real-time data and changing epidemiological information could transform health care in low-income countries by using existing and emerging data sources and data-management technologies to drive logical and potentially self-learning decision-making algorithms.
2. A DCA system will require an accurate means of unique patient identification (ideally, through biometrics), digitized medical and health data, and a connection with supervisory, referral, and policy levels of the health care system.
3. DCA software development will require the expertise of a multidisciplinary team of clinical, policy, public health, communication, and data engineering experts. However, there is no technology gap preventing such an evidence-based system from impacting health worker practice.
